# Nanoparticles with Ampholytic Surfaces for Binding and Disintegration of Amyloid Fibrils

**DOI:** 10.1021/acscentsci.5c00519

**Published:** 2025-07-02

**Authors:** Suman Mandal, Minh Dang Nguyen, Nikhil Ranjan Jana, T. Randall Lee

**Affiliations:** † Department of Chemistry and the Texas Center for Superconductivity, 14743 University of Houston, Houston, Texas 77204-5003, United States; ‡ School of Materials Science, 62397 Indian Association for the Cultivation of Science, Kolkata 700 032, India

## Abstract

Amyloid fibrils and associated protein aggregates are key contributors to a range of neurodegenerative diseases. Recent studies suggest that nanoparticles with tailored surface chemistries can effectively bind to and disrupt these fibrils. Here, we investigate the role of nanoparticle surface charge in mediating interactions with amyloid fibrils and promoting their disintegration. We synthesized seven types of charged iron oxide nanoparticles (cationic, anionic, and ampholytic) in colloidal form with hydrodynamic diameters ranging from 15 to 40 nm. Interaction studies with mature lysozyme fibrils revealed that ampholytic nanoparticles exhibited the highest binding affinity among the tested surface types. This enhanced affinity is attributed to reduced nonspecific interactions and favorable electrostatic compatibility. Ampholytic nanoparticles disrupted mature amyloid fibrils approximately 2.5 times more effectively than other surface-charged nanoparticles, leading to smaller fibril fragments via mechanical agitation. We further show that agitation-induced mechanical force, along with piezocatalytically generated reactive oxygen species (ROS), contributes to fibril degradation. These findings highlight the critical role of ampholytic surface charge in promoting fibril disintegration and suggest that such nanoparticles could be leveraged in therapeutic strategies for neurodegenerative diseases involving amyloid aggregation.

## Graphical abstract

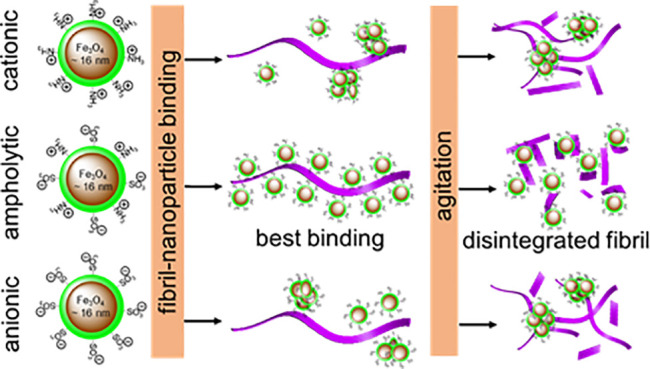

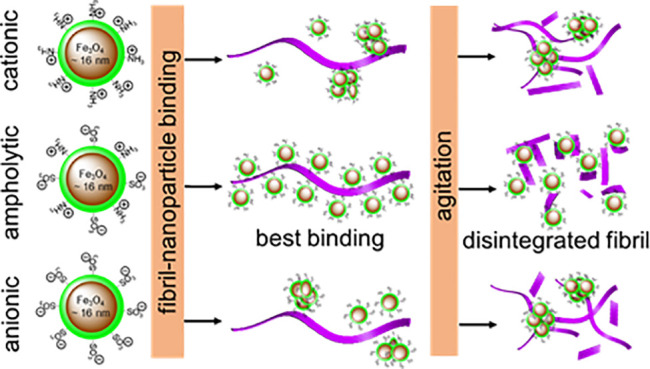

## Introduction

Numerous human diseases are associated with the formation of insoluble protein aggregates commonly called amyloid fibrils or plaques.^1^ Soluble proteins undergo structural alterations and form such aggregates that accumulate in cells, tissues, and organs.^1,2^ Amyloid fibrils are implicated in neurodegenerative diseases such as Alzheimer’s, Parkinson’s, Huntington’s, and various forms of amyloidosis.^3^ In general, two processes contribute to the formation of amyloid fibrils in vivo. One is linked to genetic disorders that introduce destabilizing amino acid residues, rendering proteins susceptible to aggregation. Another process involves elevated protein concentrations due to either overexpression or impaired protein breakdown, leading to nucleation and formation of toxic protein aggregates (oligomers and fibrils).^4^ Alzheimer’s disease (AD) falls into the latter category and stands as the most prevalent neurodegenerative disorder characterized by the abnormal accumulation of amyloid β-peptides outside neuronal cell membranes.^3,4^ These peptides, primarily Aβ-40 and Aβ-42, tend to form oligomeric and fibrillar aggregates. These aggregates give rise to the development of amyloid plaques (outside the neurons) and neurofibrillary tangles (formation of abnormal clumps due to tau protein aggregation inside the neurons) in the brain, leading to neuronal loss.^3^ The deposition of amyloid β outside neuronal cells disrupts intercellular signaling. Over time, these aggregates interact with cell membranes or enter cells, triggering intracellular pathogenesis and ultimately leading to neuronal loss.^3^ Thus, the primary therapeutic approach against AD focuses on targeting and clearing amyloid β.^5–9^ While currently approved drugs provide only symptomatic relief, the recent FDA approval of aducanumab (marketed as “Aduhelm”) represents a significant milestone as the first treatment addressing the core pathophysiology of AD since 2003.^8–11^ However, concerns persist regarding its efficacy and high cost. Therefore, ongoing research is focused on developing new drugs and cost-effective strategies to target and degrade amyloid β aggregates.^6,7,12–14^

Until now, nanotechnology-based approaches have largely utilized various types of nanoparticles to prevent fibril aggregation or break down matured fibrils in the extracellular space.^15^ It has also been shown that nanoparticle forms of anti-amyloidogenic small molecules such as polyphenols, amino acids, and sugars offer enhanced performance in inhibiting amyloid aggregation in vitro.^12,15–19^ However, no nanoparticles have been found to influence the fundamental pathophysiology of Alzheimer’s disease in vivo. However, recent nanotechnology-based approaches have shown promise in eliminating amyloid β aggregates from the brain.^6,7,14^ It has also been demonstrated that targeting fibrils with nanoparticles induces autophagy, a cellular self-clearing process.^6^ These research findings have motivated the development of nanoparticles that can effectively bind to fibrils, potentially opening doors for both treatment and diagnosis. The surface chemistry of nanoparticles might play a crucial role in this endeavor mainly for three reasons: first, it offers water dispersibility of nanoparticles; second, it can facilitate interaction with biological entities; and third, it offers biocompatibility and biodegradability.^12,15,18^

In this work, we designed several Fe_3_O_4_ nanoparticles (IONPs) having core diameters of ∼7 and ∼16 nm with varied charge and surface functionality to evaluate their potential interactions with lysozyme amyloid fibrils (LFs) made from lysozyme protein. We applied a polyacrylate coating to obtain water-soluble and colloidally stable IONPs; the coating also offers the opportunity to explore the surface charge/functionality. We extensively used transmission electron microscopy (TEM) to explore the best suitable surface composition that offers the strongest interaction with the amyloid fibrils. The results indicated that the ampholytic surface composition, having −SO_3_H as anionic and −NH_2_ as cationic groups, attached to the fibril surface efficiently. The ampholytic nanoparticles were found to attach quickly on the surface of the fibrils and form a homogeneous nanoparticle–fibril composite. Furthermore, applying external agitation to the preformed LFs in the presence of the ampholytic nanoparticles facilitated the disintegration of the LFs efficiently by mechanical force and piezocatalytically generated reactive oxygen species (ROS)-based oxidative degradation (Scheme 1). We believe that this resourceful surface chemistry study will help design future nanomedicines as detecting/targeting agents in vitro/in vivo and that the small water-soluble IONPs have the potential to offer magnetic hyperthermia-based AD treatments.

**Scheme 1:**
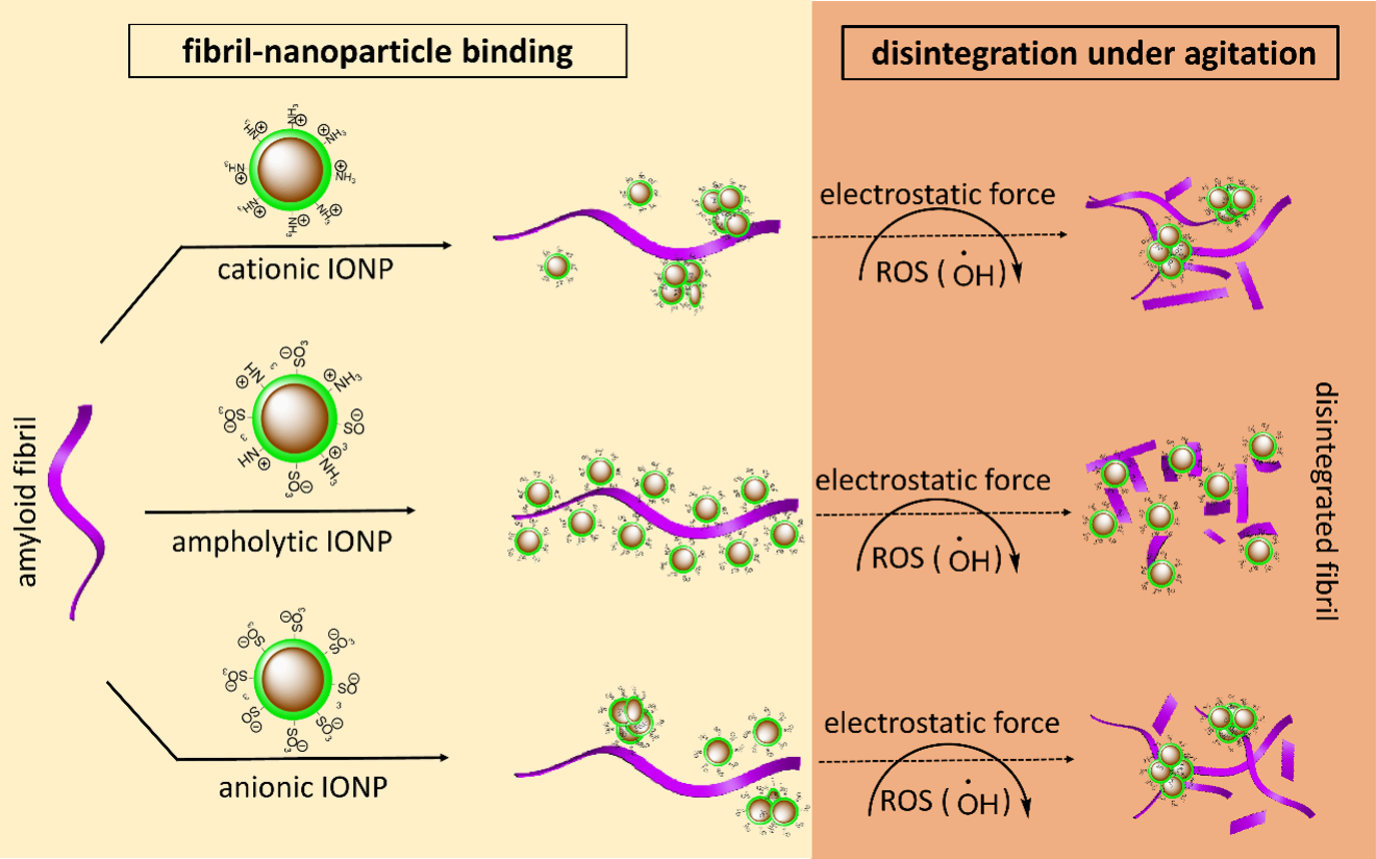
Surface Chemistry-Dependent Nanopaticle-Amyloid Fibril Binding and Disintegration under Agitation^a^

a Nanoparticles with an ampholytic surface (having both positive and negative charges) more effectively interact with amyloid fibrils, destabilizing and fragmenting them through a combination of attractive and repulsive forces and generating reactive oxygen species during mechanical agitation that aid in the disintegration process. In contrast, cationic surface causes weaker fibril disruption and forms larger aggregates, while anionic surface shows limited interaction and less effective disintegration.

## Materials and Methods

### Chemicals

For the synthesis of IONPs, iron­(III) chloride hexahydrate (97%, Alfa Aesar), sodium oleate (97%, TCI), 1-hexadecene (for synthesis, Sigma-Aldrich), 1-octadecene (90%, Sigma-Aldrich), and oleic acid (90%, Sigma-Aldrich) were used as reagents. Solvents such as deionized water with a resistance of 18 MΩ-cm (Academic Milli-Q Water System, Millipore Corporation), ethanol (200 proof, Decon Laboratories) and *n*-hexane (99%, Oakwood) were used consistently for synthesis. *N*-(3-amino­propyl)­meth­acryl­amide, 3-sulfopropyl methacrylate, acrylic acid, tetramethylethylenediamine (TMEDA), IGEPAL-500, nitroblue tetrazolium (NBT), terephthalic acid, l-aspartic acid, epigallocatechin gallate (EGCG), and lysozyme powder were purchased from Sigma-Aldrich and used as received. Thioflavin T was purchased from TCI Chemicals. The buffer solutions pH 4.5 and pH 9 were purchased from Supelco. MES buffer (pH 5.5) was purchased from Bioworld. DMEM cell culture medium and GlutaMAX were purchased from Thermo Fisher Scientific. Fetal bovine serum (FBS) was purchased from Cytiva. Penicillin-streptomycin (PS) was purchased from Sigma, and Trypsin-EDTA (0.25%) was purchased from Gibco.

### Synthesis of IONPs (Hydrophobic)

The thermal decomposition of iron­(III) oleate precursors in high-boiling-point solvent was used to synthesize IONPs with some modifications.^20^ First, the iron­(III) oleate precursor was synthesized. A mixture containing 10.8 g of FeCl_3_·6H_2_O, 36.5 g of sodium oleate, 80 mL of ethanol, 60 mL of deionized water, and 140 mL of hexane was refluxed at 70 °C for 4 h. This product mixture consists of a top organic layer containing iron­(III) oleate and a bottom transparent layer containing polar components. The extraction process was carried out with 200 mL of deionized water each time and by repeating the extraction at least 5 times. Finally, to collect the iron­(III) oleate precursor, hexane was evaporated using a rotary evaporator. The iron­(III) oleate precursor was then dried in an oven at 80 °C for 48 h to ensure the removal of residual hexane. Next, iron­(III) oleate, oleic acid, and a nonpolar organic solvent (1-hexadecene or 1-octadecene) was mixed thoroughly and degassed with N_2_ for 2 h, followed by refluxing at 280 °C (1-hexadecene solvent) or 310 °C (1-octadecene) for 30 min while maintaining N_2_ bubbling throughout the reaction. Conditions for the synthesis of the 7 and 16 nm IONPs are specified in Table 1.

**Table 1:** Chemical Synthesis Conditions for Iron Oxide Nanoparticles

Iron(III) Oleate (g)	Oleic Acid (g)	Solvent (g)	Temperature (°C)	Diameter (nm)
1.8	0.285	12.57	280 °C	7
2.2	0.300	12.57	310 °C	16

The nanoparticles were separated from solution by centrifugation after the addition of ethanol to precipitate the nanoparticles. The supernatant was then removed, and the nanoparticles were redispersed in hexane. The washing cycle was repeated 3 times, and the nanoparticles were dispersed in hexane for preservation.

### Phase Transfer and Surface Functionalization of IONPs

Hydrophobic IONPs were synthesized having different sizes, and they were subsequently converted into water-soluble, polymer-coated nanoparticles via in situ polymerization of acrylate/acrylamide monomers.^21^ Hydrophobic IONPs (1.5 mg/mL) were purified from free surfactants and then dissolved in reverse micelles. Next, the solution was transferred to a three-neck flask, mixed with the monomers, and purged with nitrogen before persulfate was added to initiate the polymerization. Three types of acrylate monomers have been used for the polymer coating: (1) acrylic acid (2 μL), (2) 3-sulfopropyl methacrylate (6.2 mg), and (3) *N*-(3-amino­propyl)­meth­acryl­amide (4.5 mg). The monomers gave rise to polymer-coated nanoparticles having carboxylic acid/sulfate/amine groups on their surfaces. For the mixed-acrylate coating, two different monomers in a 1:1 ratio were used, keeping all other conditions the same. After polymerization, IONPs were separated from the reverse micelles, dissolved in water, and dialyzed against distilled water (using a membrane with a molecular weight cutoff of 12 kDa) to remove free reactants/polymers.

### Synthesis of Ampholytic Quantum Dots (QDs) and Ampholytic Polyaspartic Acid (PAA) Nanoparticles

The hydrophobic QD was synthesized and further coated with acrylate monomers following our previously reported method with some modifications.^21^ Polyaspartic acid was synthesized using a previously reported method with some modifications.^22^ In brief, 3 g of l-aspartic acid was dissolved in 7 mL of mesitylene solvent, and 165 μL of 88% phosphoric acid was added to the suspension. This mixture was then heated at 150 °C for 4–5 h under an argon atmosphere. After the mixture was cooled to room temperature, 35 mL of DMF was added to dissolve the product, followed by the addition of excess water to reprecipitate the polymer. The obtained polymer was washed multiple times with water and methanol and then dried under vacuum. Next, the functionalized polymeric carrier was synthesized by reacting polyaspartic acid with oleylamine and ethylene diamine at 80 °C. The functionalized polyaspartic acid (PAA) was then dissolved in DMSO, and micelles were formed in water. The micelles were purified by dialysis and stored at 4 °C for further use.

### Amyloid Fibril Preparation Using Hen Egg White Lysozyme (HEWL) and Aβ (1–42) Peptide

A HEWL solution of approximately 1 mL (1 mg/mL, 70 μM) was prepared by dissolving HEWL in water with acetate buffer at pH 5.0 (adjusted with HCl), along with the addition of NaCl (137 mM) and KCl (2.7 mM).^18^ This protein solution was heated to 57 °C and stirred for 12 h. For the preparation of Aβ fibrils, Aβ peptide solution was prepared using a standardized hexafluoro-isopropanol pretreatment, followed by incubation in PBS at 37 °C for 72 h under agitation. The resulting fibrils were subsequently purified by using two distinct methods. In one procedure, a portion of the reaction mixture was subjected to centrifugation at 2000 rpm, and the precipitate was then redispersed in fresh deionized water. This process was repeated, and the final product was redispersed in 1 mL of water. In the other approach, the mixture was enclosed within a dialysis membrane (with a molecular weight cutoff of 12 kDa) and dialyzed against water for a period of 24 h, after which the amyloid fibrils were collected. Subsequently, the fibrils were visualized under a TEM, and their physicochemical properties were assessed using a Malvern Zetasizer Nano instrument.

### Fibril–Nanoparticle Interaction Study

To a preformed amyloid fibril solution (final concentration of 0.1 mg/mL) dispersed in PBS pH 7.4, an aqueous solution of IONPs was added (final concentration of 10 μg/mL), and the mixture was incubated for 15 min at 37 °C and kept undisturbed. A 20 μL aliquot was taken, diluted, drop-cast onto a copper grid, and imaged by TEM. Further, we used a modified drop-casting procedure to minimize any potential reorganization during the drying on the TEM grid. We allowed the droplet of aqueous nanoparticle solution to remain undisturbed before carefully removing the solution with a micropipette. This process helps ensure that the nanoparticles bound to the fibrils remain in place, while unattached nanoparticles are discarded.

### Fibril Disintegration Study in the Presence of Nanoparticles

Mature amyloid fibrils were prepared from HEWL by the standard conditions as described above, along with the addition of acid (∼2 μL HCl) to catalyze the fibrillation. Fibrils were purified by dialysis, followed by redispersion in PBS buffer (pH 7.4). Next, dispersed fibrils (0.1 mg/mL) were incubated with IONPs at 37 °C for 1 week under agitation. The kinetics of disintegration were monitored using the thioflavin T assay.^23^ Typically, 10 μL samples of the protein solution were collected at various time intervals and mixed with 1 mL of 10 μM thioflavin T solution in buffer solution (PBS pH 7.4). After 5 min, the thioflavin T fluorescence was measured at 485 nm with 440 nm excitation. To compare the impact of nanoparticles on the disintegration kinetics, one control set without any nanoparticles was assessed by the same process. After the completion of the experiment, circular dichroism (CD) spectra were obtained, and TEM measurements were performed. For the ultrasound treatment, the entire reaction mixture containing fibrils was exposed to ultrasound vibration (1 MHz, 1.5 W cm^–2^, 50% duty cycle) for 1 h.

### Detection of ROS during Fibril Disintegration

Reactive species during fibril disintegration were detected by using nitroblue tetrazolium (NBT) as a probe for superoxide anion detection. In this assay, NBT was used at a final concentration of 46 μM and mixed with a 20 μL aliquot from the reaction mixture. The solution was incubated in the dark for 30 min, after which the absorbance of NBT at 259 nm was measured by using a UV–vis spectrophotometer. To detect hydroxyl radicals, terephthalic acid was employed as a probe, as it reacts with hydroxyl radicals to form hydroxylated terephthalic acid, which exhibits fluorescence at 430 nm when excited at 315 nm. In this case, 20 μL of 0.5 mM terephthalic acid in PBS pH 7.4 was mixed with 20 μL of the reaction mixture and vortexed in the dark, and fluorescence was measured using a fluorescence spectrophotometer.

### Instrumentations

All synthesized IONPs and nanoparticle–fibril interactions were imaged by a JEOL JEM-2010FX TEM operating at 200 kV. All samples for TEM characterization were deposited on 300 mesh holey carbon-coated copper grids and dried overnight at room temperature before analysis. The UV–vis absorption spectra of samples were collected using a Shimadzu UV-2550 UV–vis spectrophotometer. Hydrodynamic diameter and zeta potential measurements were conducted on a Malvern Zetasizer model ZEN3600 instrument. Thermogravimetric analysis (TGA) was carried out on a TA SDT Q600 instrument at a constant heating rate of 10 °C/min. Emission spectra were recorded using a PerkinElmer LS 55 fluorimeter. CD spectra were measured using a CD spectrometer (Jasco, model J-815-1508). An Intelect Mobile 2 Ultrasound instrument (Chattanooga, USA, with 1 and 3 MHz frequencies and 0.5–3 W cm^–2^ power) was used as an ultrasound source.

## Results and Discussion

### Water-Soluble IONPs with Varied Surface Chemistry

We designed Fe_3_O_4_ nanoparticles with different surface compositions (functional groups). IONPs were selected because they are widely used as MRI contrast agents and in magnetic hyperthermia therapy, ferroptosis therapy, and various other biomedical applications.^24,25^ The surface composition is important because it can modulate colloidal stability, nano–bio interactions, and increase cellular uptake via predominant endocytosis with low endosomal/lysosomal trafficking.^21^ The synthesis involved the transformation of hydrophobic IONPs (7 nm and 16 nm) nanoparticles to polyacrylate-coated water-soluble nanoparticles with a 30–40 nm hydrodynamic size. The surfactant-capped hydrophobic IONPs were converted to hydrophilic IONPs with the desired surface chemistry via a reverse microemulsion-based polyacrylate coating. A variety of acrylate monomers were used during the polyacrylate coating, and the chemical structures of the respective acrylate monomers are shown in Scheme 2. We used a reverse microemulsion-based phase transfer and surface polymer coating approach (depicted in the Supporting Information, Scheme S1). The hydrophobic IONPs capped with oleic acid were well dispersed in cyclohexane solution, in which the ligands were rapidly exchanged and then inserted inside the reverse micelle core. Within the reverse micelle core, the polyacrylate polymerization occurred on the surface of the IONPs, initiated by ammonium persulfate, and gradually covered the whole nanoparticle to form a polymer shell. The coating time was maintained for 2 h even though 20–30 min is usually sufficient to ensure the entire consumption of the acrylate monomers to form the shell. The acrylate monomers shown in Scheme 2, with appropriate molar ratios, were used for polyacrylate coatings that provide colloidal stability to the nanoparticles with modulated surface charges. The *N*-(3-amino­propyl)­meth­acryl­amide monomer offers positive charges, and the 3-sulfopropyl methacrylate and acrylic acid monomers offer negative charges at physiological pH 7.4. Depending on the surface charge (coming from the polyacrylate composition used), IONPs are named as follows: CAT 1 (positively charge), AMP 1 (neutral charge), and ANI 1 (negative charge).

**Scheme 2:**
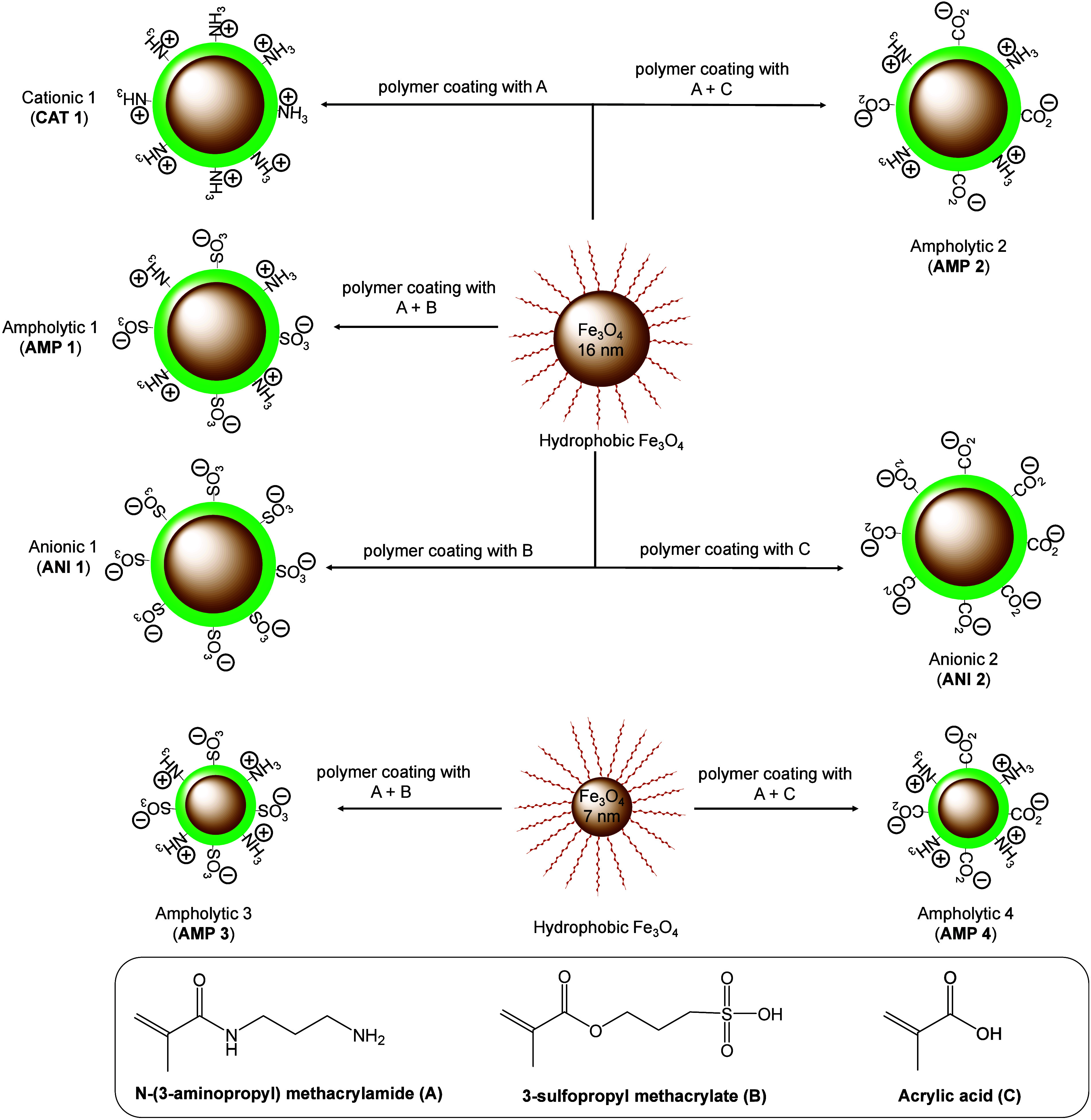
IONPs with Diverse Surface Chemistries^a^

a A polyacrylate coating renders the nanoparticles water-soluble. Employing various acrylate monomers allows for modulation of the surface chemistry and charge. Acrylates terminated with amine groups impart a cationic character, while those terminated with carboxylate or sulfopropyl groups impart an anionic character. Combining these two monomers in a 1:1 ratio yields nanoparticles with balanced charge, which are referred to as ampholytic nanoparticles.

The physicochemical properties of all surface-modified IONPs are listed in Table 2. Hydrodynamic diameters of the functionalized nanoparticles varied from 20 to 50 nm (for the 16 ± 2 nm core) at physiological pH (7.4). The hydrodynamic diameters in PBS (pH 7.4) are relatively larger than those of the core IONPs due to the formation of large charge double layers (Figure 1a). The ampholytic and anionic nanoparticles showed relatively larger hydration spheres. The increased hydrodynamic diameter of ampholytic and anionic nanoparticles is presumably due to the low surface charge that induce particle–particle agglomeration. In particular, the agglomeration of ampholytic nanoparticles occurs due to particle–particle interaction, as each particle has both cationic and anionic charges. Thermogravimetric analysis showed that there was a sharp decrease in wt % after 200 °C, which can be attributed to the loss of the polymer surface coating, and the graph indicates a ∼10–15 wt % polymer coating (Figure 1b).

**Table 2:** Physicochemical Properties of Designed Water-Soluble IONPs^b^

Sample	Core Diameter (nm)	Acrylate Monomer Used^a^	Hydrodynamic Diameter (nm)	Zeta Potential (mV)	Functional Groups	Colloidal Stability
CAT 1	16 ± 2	A	20 ± 5	+10 ± 2	NH_2_	>2 months
AMP 1	16 ± 2	A:B (1:1)	40 ± 10	–2 ± 1	NH_2_, SO_3_^–^	>2 months
ANI 1	16 ± 2	B	40 ± 10	–7 ± 2	SO_3_^–^	>2 months
AMP 2	16 ± 2	A:C (1:1)	25 ± 10	–1 ± 3	NH_2_, COOH	>2 months
ANI 2	16 ± 2	C	25 ± 5	–9 ± 2	COOH	>2 months
AMP 3	7 ± 2	A:B (1:1)	15 ± 3	+1 ± 1	NH_2_, SO_3_^–^	>3 months
AMP 4	7 ± 2	A:C (1:1)	15 ± 5	+1 ± 3	NH_2_, COOH	>3 months

a Hydrodynamic size, zeta potential, and colloidal stability for nanoparticles are measured in PBS pH 7.4.

b A, *N*-(3-amino­propyl)­meth­acryl­amide; B, 3-sulfopropyl methacrylate; C, acrylic acid.

**Figure 1:**
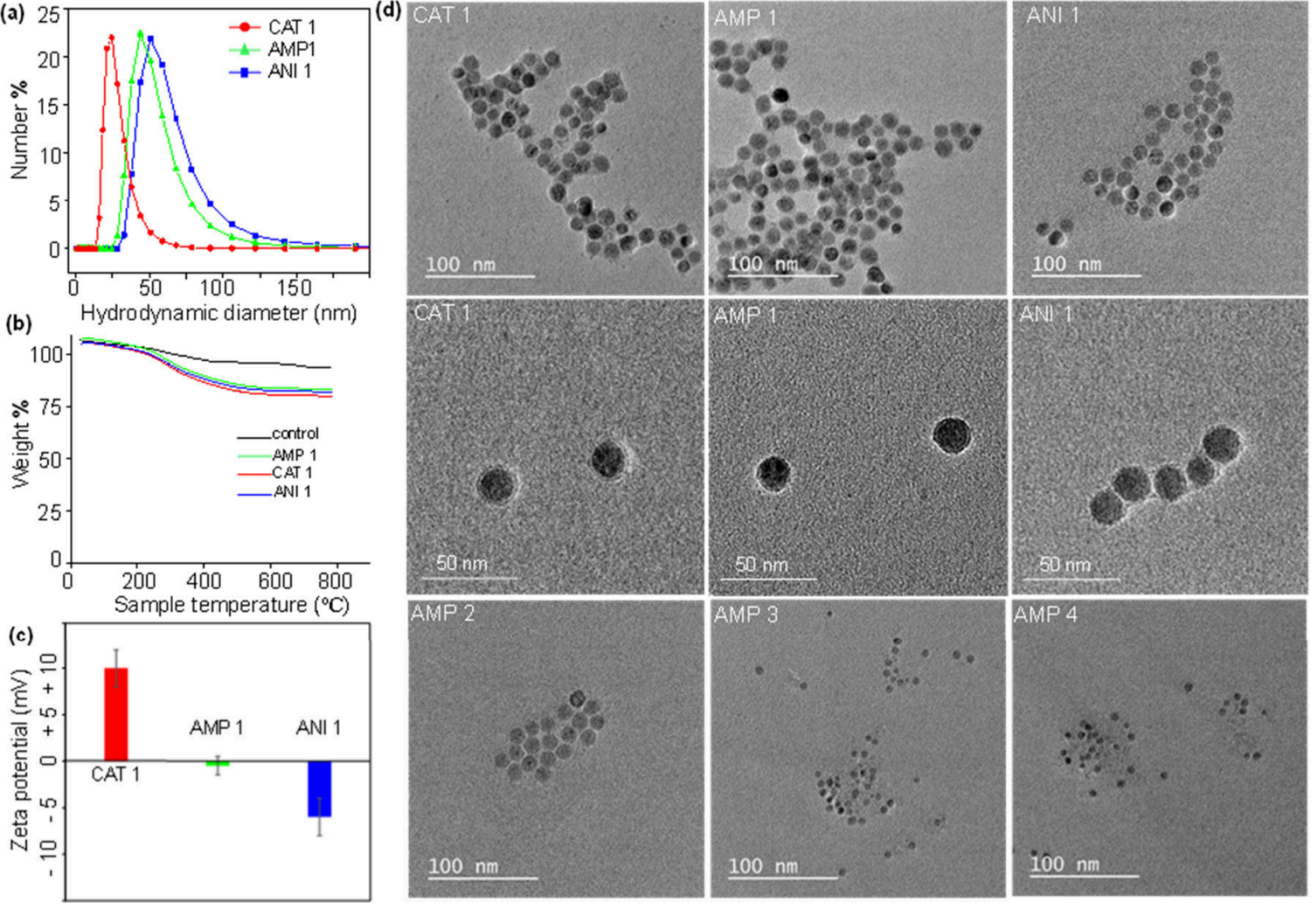
(a) Hydrodynamic diameter distribution of the IONPs measured in PBS (pH 7.4). (b) Thermogravimetric analysis displaying the weight percentage of the polymer coating on nanoparticle surfaces. (c) Zeta potentials measured in PBS (Ph 7.4), revealing three distinct surface charges of nanoparticles ranging from cationic to neutral to anionic. (d) Images from TEM for polymer-coated water-soluble IONPs.

The surface zeta potential of the functional IONPs correlated well with the surface functionalization, showing a positive value for the CAT 1 (+10 ± 2) and a negative value for the ANI 1 (−7 ± 2), as shown in Figure 1c and Table S1. The low charge (near zero, −2 ± 2) for the AMP 1 at pH 7.4 can be attributed to the presence of primary amines (that offer positive charge after protonation) that are counterbalanced by anionic sulfopropyl groups. We term this surface coating as “ampholytic”, where we assume that the polymer coating contains an equal number of cationic and anionic parts.^26,27^ The nanoparticles showed much higher surface zeta potential in deionized water, but in the case of phosphate-buffered saline, the ions in solution mask the surface charge to some extent. TEM imaging showed that the core size of IONPs were well intact after the application of the polyacrylate coatings (Figure 1d and Supporting Information, Figure S1). The nanoparticle core is spherical in nature and homogeneous (for CAT 1, AMP 1, ANI 1, AMP 2, and ANI 2, the core diameter is ∼16 ± 2 nm, and for AMP 3 and AMP 4, the core diameter is ∼7 ± 2 nm). The good colloidal dispersibility (described later) highlights the effectiveness of the polymer coating. The TEM images also reveal the presence of a thin polymer shell associated with the core (low contrast with respect to the Fe_3_O_4_ core) (Figure 1d), which highlights the effectiveness of this coating approach and the polymer compositions. The Fe_3_O_4_ core is hard in nature, but the polymer coating around the nanoparticles makes them soft and more biocompatible.^26,27^

The colloidal stability of all the IONPs is excellent in water, as seen from the digital images taken after 1 month (Supporting Information, Figure S2a). A separate study showed that CAT 1, AMP 1, and ANI 1 IONPs are also stable in PBS (pH 7.4). Ampholytic nanoparticles did not precipitate readily in salt concentrations (salt out effect); only visible aggregation of the nanoparticles was seen after 1 day in the case of high salt concentrations (Supporting Information, Figure S2b).

While salt concentrations (>0.5 M) induce agglomeration (as shown in Figure S2c), subsequent sonication results in a clear and stable solution for over a week. The agglomeration is due to particle–particle interaction that is expected, as each particle has both cationic and anionic charges. These results suggest not only the colloidal stability but also the long-term stability of the polymer coating. There are at least three distinct advantages to these polymer-coated IONPs. First, the functional nanoparticles are water-soluble with good colloidal stability at physiological pH (pH 7.4). The colloidal form of nanoparticles allows their accessibility and interaction with proteins and amyloids.^15^ Second, the nanoparticles have a surface with moderate to low surface charge that is suitable for minimizing nonspecific interactions with any biointerface.^21,26,27^ Even for the ampholytic nanoparticles, low surface charge is ensured by an appropriate balance of cationic and anionic functional groups on the particle surface. Compared to nonionic surface charges, this type of poly-ampholytic surface with localized charge domains is ideal for nano–bio interactions without appreciable cytotoxicity.^27^ Third, the polymer coating offers functional groups (amine/acid) that can be used to further conjugate with specifically targeted biomolecules and ligands.^21^

### Nanoparticles with Ampholytic Surfaces Strongly Interact with Amyloid (Lysozyme) Fibrils

We have extensively used HEWL as an in vitro model protein for synthesizing amyloid fibrils and studying interactions with the nanoparticles, as it shows ultrastructures and its biochemical properties are similar to those of pathological deposits in tissue.^23,28^ Amyloid fibrils formed from this protein are not associated with any known amyloid diseases, but they share morphological features similar to those of amyloid fibrils from disease-associated proteins and can be inherently highly cytotoxic. Studies of amyloid aggregation of nondisease-associated proteins not only aid in understanding the mechanism of amyloid fibrillogenesis but also extend our understanding of the basic relationship between protein sequence and structure. Moreover, lysozyme is a common model protein for studying protein fibrillation in extracellular space. A common strategy to convert a nondisease-associated protein to amyloid fibrils is destabilizing the protein either by mutation or by partial denaturation with heating or the addition of acids/salts.^29^ Incubation of lysozyme in an acidic medium at elevated temperature led to the formation of amyloid fibrils. Typically, we incubated 1 mg/mL lysozyme in 150 mM concentrated sodium chloride solution at 57 °C under stirring. The fibrils were purified using two methods: (1) centrifugation-redispersion and (2) dialysis (Figure 2). The first method is widely used, as it gives mature fibrils having network-like distributions. However, centrifugation leads to fibril aggregation, which creates problems in obtaining individual fibrils. To minimize these effects, we introduced the technique of dialysis. Dialysis can remove the attached unwanted ions (e.g., Na^+^, K^+^, Cl^–^) to give deprotonated fibrils. We studied both fibril types under TEM with no significant change in their length (1–5 μM) or distribution after redispersion. Notably, the surface zeta potential shifts slightly negatively after dialysis (−2 ± 2) compared to that of the precipitated one (+4 ± 2) (Table S2).

**Figure 2:**
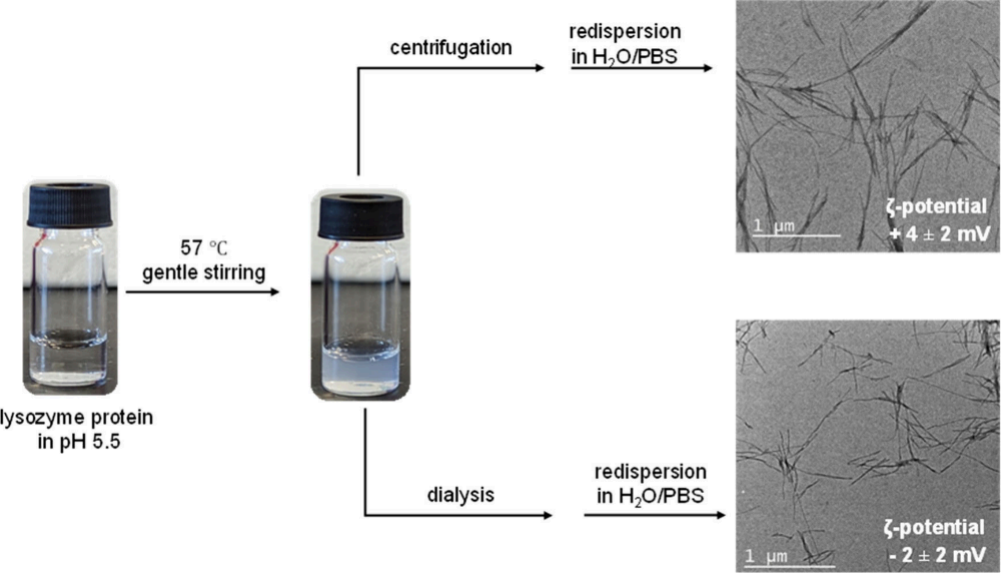
Synthesis and purification of lysozyme fibrils, as well as TEM images of the fibrils obtained by using two distinct purification methods. The insets provide information about the zeta potentials at pH 7.4.

Next, we used both types of fibrils and studied their interaction with the synthesized IONPs in the solution phase. The interaction between nanoparticles and amyloid fibrils is a critical area of study due to its implications for both nanotechnology and neurodegenerative disease research. Studies have shown that nanoparticles can either inhibit or accelerate fibril formation depending on their composition, size, and surface charge.^30–35^ Several investigations have shown that IONPs (surface modified/unmodified) have anti-amyloidogenic properties, but there is no clear evidence on what type of surface is necessary for that.^36–42^ Here, we extensively studied IONPs vs fibril interactions at the extracellular level to explore their potential (Figure 3). Nanoparticles (10 μg/mL) and the preformed (purified by dialysis method) fibrils (0.1 mg/mL) were incubated at 37 °C in PBS pH 7.4 buffer for 15 min and then imaged by TEM. The CAT 1 nanoparticles attached to the fibrils, but they appeared aggregated; similarly, the ANI 1 nanoparticles showed little interaction with the fibrils. Instead, they likely formed clans with other ANI 1 nanoparticles. In contrast, the AMP 1 nanoparticles mostly attached to the surface of the fibrils (Figure 3a). Figure 3b (higher magnification) shows more clearly how the AMP 1 nanoparticles are decorated along the surface of the fibrils. The other ampholytic nanoparticle (AMP 2) also showed good attachment with the fibrils but not the ANI 2 nanoparticles, which aggregated with themselves rather than the fibrils (Figure 3c). All of these nanoparticles are ionic, and they have a magnetic core (∼16 nm Fe_3_O_4_), so particle–particle aggregation is hard to restrict. In this regard, ampholytic nanoparticles should be more prone to aggregation, as they have complementary surface charges toward each other. Surprisingly, the ampholytic surfaces are probably playing the main role to target the fibril surface better than the singly charged particles (CAT/ANI). In the case of the AMP 1 nanoparticles vs fibrils, as most of the nanoparticles are attached to the fibrils, there is no visible nanoparticle aggregation. We performed TEM imaging using a modified drop-casting method to assess potential reorganization during the drying process. The TEM images indicate no significant visible nanoparticle–fibril binding when fibrils were incubated with CAT 1 or ANI 1 nanoparticles, whereas AMP 1 nanoparticles exhibit a similar tendency to attach to the fibrils effectively (Figure S3a). To further validate our observations, we performed ICP-MS analysis. We incubated nanoparticles and fibrils; after 10 min, we centrifuged the solution at 1000 rpm, took an aliquot from the supernatant, and processed it for ICP-MS measurements (Supporting Information, Figure S3b). Our results demonstrate that the supernatant of ampholytic nanoparticles contains the least iron, suggesting that these nanoparticles are more effectively bound to the fibrils. In contrast, the supernatant of anionic nanoparticles contains the highest iron concentration, indicating less binding to the fibrils (Figure S3c).

**Figure 3:**
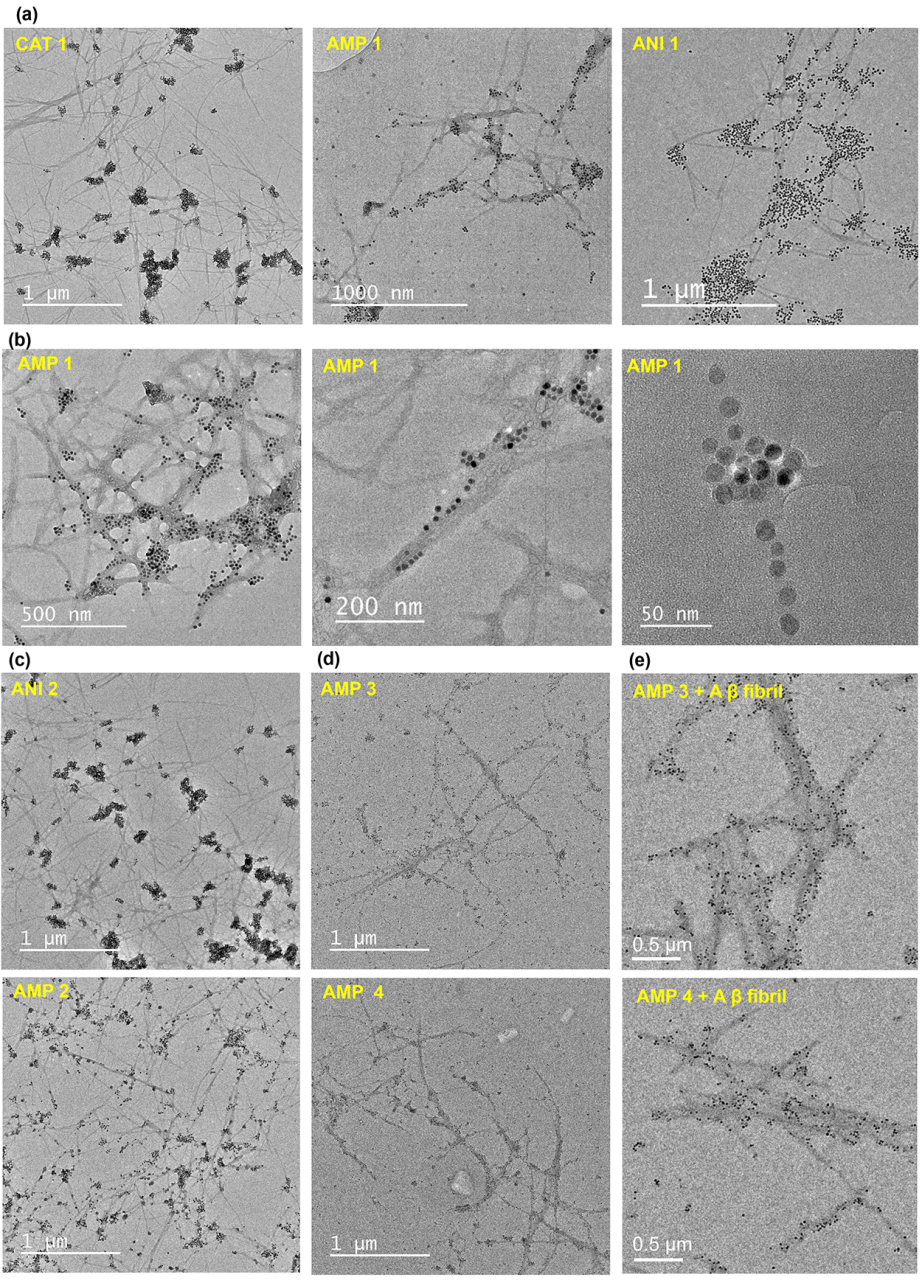
Lysozyme amyloid fibril (LF) binding study of functional IONPs observed by TEM. (a) LF vs CAT 1/AMP 1/ANI 1, high magnification images of (b) LF vs AMP 1, (c) LF vs ANI 2/AMP 2 (d) LF vs AMP 3/AMP 4 (having a ∼7 ± 2 nm core), and (e) Aβ fibril vs AMP 3/AMP 4 NPs.

This further supports the interpretation that ampholytic nanoparticles preferentially interact with amyloid fibrils in solution. The same study performed using the fibrils purified by the precipitation-redispersion method showed similar tendencies for the CAT 1, AMP 1, and ANI 1 nanoparticles (Supporting Information, Figure S4a). The AMP 2 nanoparticles (having carboxylate as the anionic group) showed attachment with the fibrils, but it was not as good as that observed for the AMP 1 nanoparticles (having sulfopropyl as the anionic group) (Supporting Information, Figure S4b). The success of the ampholytic surface composition was also found with the smaller (∼7 nm) Fe_3_O_4_ core (AMP 3 and AMP 4). As shown in Figure 3d, the smaller ampholytic nanoparticles are even more closely decorated with the fibrils and distribute homogeneously along the fibril surface, resembling a fibril–nanoparticle composite. It is also evident that the smaller ampholytic IONPs target the fibrils even more efficiently and show no significant particle–particle aggregation. We have applied the same targeting approach on amyloid β fibrils (made from Aβ-42 peptides), and the TEM images reveal that the ampholytic nanoparticles bind well on the Aβ fibril surfaces (Figure 3e). While lysozyme fibrils were used as a model system throughout this study, it is important to note that amyloid β fibrils are known to exhibit polymorphism, which can influence their biochemical behavior and interaction with nanomaterials. While polymorphism of lysozyme fibrils is not well-established, we selected this model system due to its well-characterized morphology and reproducibility in vitro.

To evaluate the selectivity of the surface-modified nanoparticles for amyloid fibrils over other extracellular matrix proteins, we performed binding studies using lysozyme fibrils (LFs) in DMEM culture medium, which simulates a protein-rich extracellular environment. The fibrils were incubated with three types of nanoparticles (AMP 1, CAT 1, and ANI 1) for 15 min, followed by low-speed centrifugation (1000 rpm). The resulting pellet was redispersed in water and analyzed using TEM. The TEM images (Supporting Information, Figure S5) revealed that all three types of nanoparticles interacted with the fibrils; however, the ampholytic nanoparticles (AMP 1) exhibited comparatively stronger and more consistent binding. The presence of nanoparticles in both the pellet and supernatant further supports that while selective binding to fibrils is evident, especially with AMP 1, interactions with other extracellular proteins cannot be fully ruled out.

To investigate whether the ampholytic surface chemistry alone governs amyloid fibril targeting, independent of the nanoparticle core, we evaluated multiple nanostructures with different core compositions but similar surface functionalities. Polyacrylate-coated amphiphilic QDs, bearing both −COOH and −NH_2_ groups similar to AMP 2, and polyaspartic acid-based polymer nanoparticles (∼50 ± 10 nm in diameter) were synthesized and examined for fibril binding (Supporting Information, Figure S6a,b). TEM images confirmed that both nanoparticle types readily associate with lysozyme fibrils (LFs), suggesting that the ampholytic surface chemistry plays a dominant role in mediating fibril interaction, irrespective of the nanoparticle’s core structure. These results emphasize the broader applicability of ampholytic surface modifications.

Previous studies have demonstrated that amyloid fibrils exhibit stronger interactions with bioentities possessing polyelectrolytic characteristics, with the strength of the interaction being dependent on the charge of the entities.^43,44^ Although amyloid fibrils display low negative surface charges, they also feature regions of local charge asymmetry, which could be complementary to the ampholytic nature of our nanoparticles. This charge complementarity is likely what allows our ampholytic-coated nanoparticles to interact more effectively with amyloid fibrils.^45^ In contrast, charged nanoparticles (either cationic or anionic) lack such a complementarity, which leads to poor binding with fibrils. However, it is important to note that these ampholytic nanoparticles can interact with other proteins in the extracellular matrix, as their surface properties allow for interactions with any protein, without showing significant cytotoxicity (Supporting Information, Figure S7). In order to enhance selectivity and minimize off-target effects, conjugation with specific targeting ligands can be implemented.^6,7^

### Ampholytic Nanoparticles Disintegrate Matured Amyloid Fibrils under Agitation

Nanoparticles disintegrate amyloid fibrils through a combination of physical and chemical interactions that destabilize their structures.^15,18,46,47^ Nanoparticles adsorb onto fibril surfaces, altering the local environment and disrupting structural integrity.^48^ Mechanical agitation can further enhance this process by applying shear forces that break apart the fibrils.^49–51^ In this study, we examined which surface charge and functionalization on the nanoparticle surface can introduce additional destabilizing interactions, such as forming complexes with fibrils and disaggregating pre-existing amyloid fibrils depending on their varied surface composition. The disintegration of amyloid fibrils by nanoparticles was quantitatively assessed using a thioflavin T (ThT) assay, which measures the fibril presence and integrity. ThT is a fluorescent dye that binds specifically to the β-sheet-rich structures of amyloid fibrils, producing a strong increase in the fluorescence. When nanoparticles interact with amyloid fibrils, they can stimulate fibril breakdown or restructuring, leading to fewer binding sites for ThT and a decrease in fluorescence intensity.^23^ By measuring fluorescence before and after nanoparticle treatment, we can determine the extent of fibril disintegration. Fibrils were generated in vitro and incubated with IONPs at a concentration of 10 μg/mL for 1 week at 37 °C. Throughout the experiment, thioflavin T (ThT) kinetics were monitored, and at the end of the incubation period, CD spectroscopy and TEM were conducted to assess structural changes. As shown in Figure 4a, the ThT signal decreased by approximately 68% for the AMP 1 nanoparticles, 30% for the CAT 1 nanoparticles, and 24% for the ANI 1 nanoparticles after 5 days of coincubation with mild agitation. These results indicate that the ampholytic AMP 1 nanoparticles were about 2 to 2.5 times more effective at disintegrating fibrils compared to the CAT 1 and ANI 1 nanoparticles. Although CAT 1 nanoparticles also exhibited some disintegration effects, AMP 1 nanoparticles demonstrated superior performance at this concentration. The ANI 1 nanoparticles, while showing some disintegration activity, were less effective than both AMP 1 and CAT 1 nanoparticles. The secondary structure of the fibrils was further analyzed using CD spectroscopy, which detects changes in protein conformation. Amyloid fibrils are characterized by a distinct CD spectrum with a strong negative peak at around 218 nm, indicative of a β-sheet-rich structure.^5^ Significant changes in the CD spectra were observed as the nanoparticles disrupted the fibrillar aggregates. A reduction or complete loss of the 218 nm peak was observed, corresponding to the breakdown of the fibrils and the loss of their organized β-sheet structure. As shown in Figure 4b, the CD spectrum of pure fibrils displayed a prominent negative peak at 218 nm, which was reduced by 50–55% following treatment with AMP 1 nanoparticles, in contrast to CAT 1 and ANI 1 nanoparticle treatments, which exhibited less pronounced changes.

**Figure 4:**
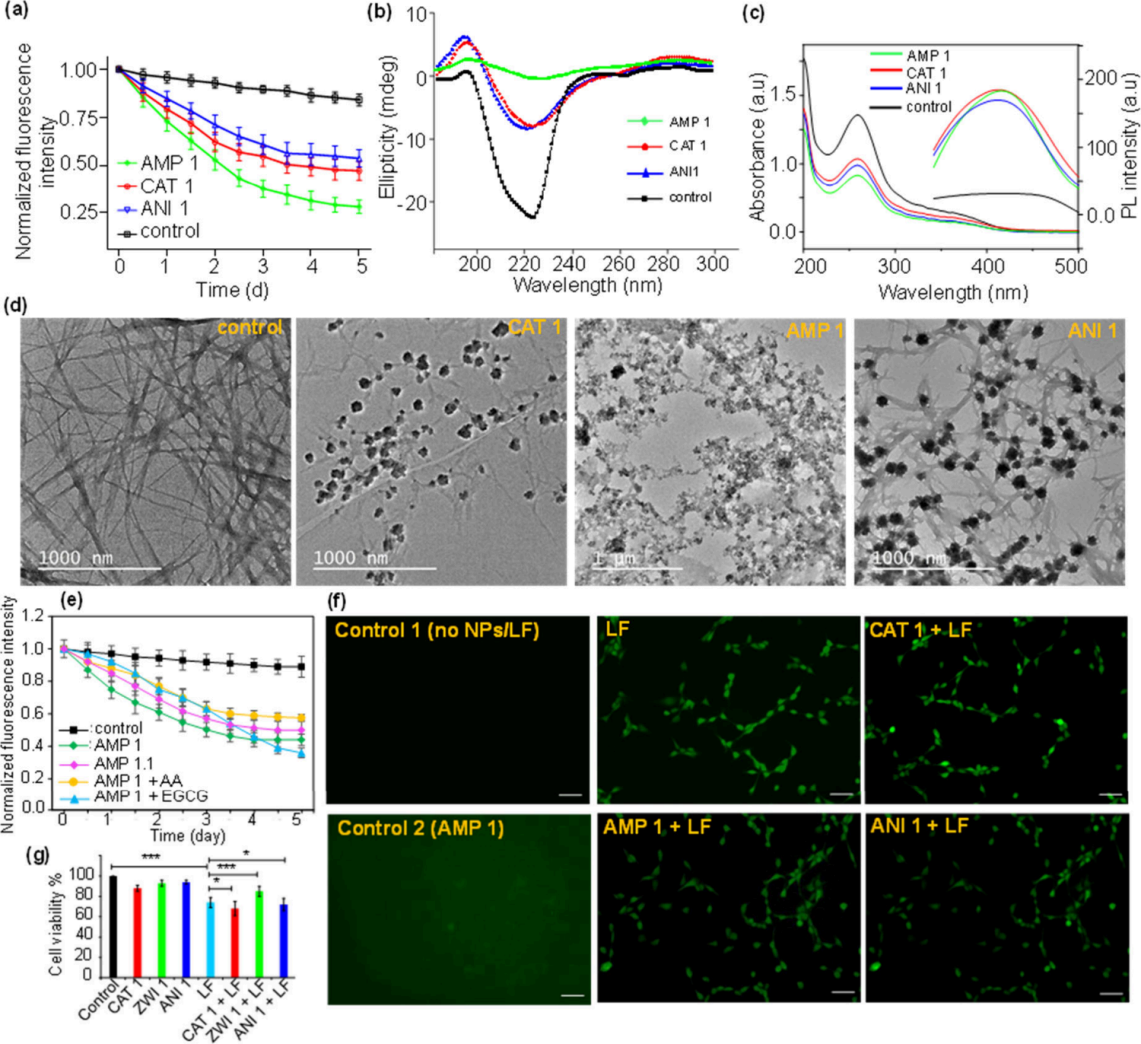
Fibril disintegration study in the presence of CAT 1, AMP 1, and ANI 1 IONPs under 5 days of agitation. (a) Thioflavin T (ThT) fluorescence assay during the fibril disintegration experiment. (b) Circular dichroism study of the fibrils after the disintegration experiment. (c) NBT assay (absorbance) and terephthalic acid assay (fluorescence) to detect the presence of reactive oxygen species during disintegration. (d) TEM images of the fibrils after 5 days of agitation in the presence of IONPs. (e) Comparative analysis of fibril disintegration efficiency between AMP 1 nanoparticles and their lower charge density variant (AMP 1.1), as well as in the presence of antioxidants ascorbic acid (AA) and epigallocatechin gallate (EGCG). (f) Intracellular reactive oxygen species (ROS) generation in HT22 cells assessed by DCF-DA staining following treatment with nanoparticles or NP–LF complexes (Scale bars: 50 μM). (g) MTT assay-based evaluation of cytotoxicity in HT22 cells exposed to nanoparticles or NP–LF complexes. Data are presented as mean ± S.D.; statistical significance: **P < 0.01, ***P < 0.001 (Tukey’s test).

We also evaluated the presence of reactive oxygen species during the disintegration process by a nitroblue tetrazolium (NBT) reduction assay and terephthalic acid (TPA) fluorescence assay (Figure 4c). The NBT assay monitors superoxide radical generation during agitation, indicated by a decrease in NBT absorbance at around 259 nm. Similarly, the TPA assay detects hydroxyl radicals by the fluorescence emitted upon their reaction with terephthalic acid. Both assays confirmed the presence of ROS during the fibril disintegration process. Interestingly, the NBT assay revealed that in the presence of CAT 1 and ANI 1 nanoparticles, ROS formation decreased as disintegration progressed (Supporting Information, Figure S8). In contrast, with AMP 1 nanoparticles, ROS levels remained constant throughout the disintegration process. This difference in ROS dynamics can plausibly be attributed to the aggregation of CAT 1 and ANI 1 nanoparticles, which might reduce their individual activity. However, no such aggregation was observed with AMP 1 nanoparticles, suggesting that they maintain their activity more effectively during fibril disintegration. TEM images of the fibrils postdisintegration further supported these findings (Figure 4d). Control experiments (no nanoparticles) showed that the fibril network remained largely intact (>2 μM length). Both CAT 1 and ANI 1 nanoparticles deformed the fibrillar structure, with CAT 1 causing extensive disruption. In contrast, AMP 1 nanoparticles not only deformed the fibrils but also promoted the formation of amorphous protein aggregates, obscuring any remaining fibrillar structure. TEM analysis also revealed that AMP 1 nanoparticles formed a homogeneous composite with the disintegrated proteins, while CAT 1 and ANI 1 nanoparticles were found as larger clumps embedded within the fibrillary network (Figure S9). These clumps were composed of individual small magnetic nanoparticles. This observation suggests that ampholytic nanoparticles interact strongly with fibrils, remaining uniformly distributed in the disintegrated protein matrix, whereas the other nanoparticles form more heterogeneous aggregates. Further control experiments involved incubating fibrils with nanoparticles for 1 week without external agitation, followed by a ThT fluorescence assay (Supporting Information, Figure S10). No significant changes in fluorescence intensity were observed for any of the nanoparticles, indicating that none of the surface modifications possessed intrinsic fibril dissolution properties under static conditions. However, after applying ultrasound treatment for 1 h, we observed a 10–15% decrease in ThT intensity for all types of IONPs. When fibrils were subsequently agitated in the presence of IONPs after 1 h of ultrasound treatment, the ThT intensity decreased by 70–80% after 2 days. This result highlights the enhanced fibril disintegration activity of IONPs under mechanical agitation accelerated by ultrasound treatment, which produces an increase in oxygen species.^51^

We have further investigated the effect of antioxidants on fibril degradation, performed in the presence of ascorbic acid and epigallocatechin gallate (EGCG), each at 10 μM, revealing a differential impact on fibril breakdown (Figure 4e). The presence of ascorbic acid or EGCG led to a modest initial reduction in fibril degradation efficiency; however, this effect diminished over time, likely due to the limited chemical stability of the antioxidants. To understand the impact of charge density on fibril disintegration, we compared the disintegration efficiency of AMP 1 nanoparticles with that of a modified variant, AMP 1.1 (with lower charge density). Thioflavin T-based fibril disintegration assays demonstrated that AMP 1.1 exhibited a ∼5% lower disintegration efficiency than AMP 1 (Figure 4e), reinforcing the notion that enhanced surface charge density facilitates stronger binding interactions with amyloid fibrils and promotes more efficient mechanical disruption. To further assess cellular oxidative stress, DCF-DA staining was performed in HT22 mouse hippocampal neuronal cells treated with nanoparticle-labeled lysozyme fibrils (NP–LF). Fluorescence microscopy showed green fluorescence in cells exposed to both free LF and NP–LF, indicating ROS generation, whereas untreated control cells exhibited no fluorescence (Figure 4f). Notably, the ROS levels in NP–LF-treated cells were comparable to those in cells treated with free LF, implying that NP conjugation does not markedly amplify ROS-induced stress. Cell viability assays (Figure 4g) further confirmed that most NP–LF conjugates are not significantly cytotoxic, with the exception of CAT 1–LF, which showed slightly reduced viability, likely due to enhanced membrane association and internalization. These results support that while ROS contribute to fibril degradation, their cellular effects remain largely manageable under the tested conditions.

Zwitterionic polymers are reported to prevent protein aggregation by stabilizing partially unfolded proteins via electrostatic and hydrophobic interaction.^53^ Here, the iron oxide-based system, especially the ampholytic variant, shows retardation of amyloid fibrillation, whereas the cationic one accelerates the lag phase and the anionic variant offers an insignificant effect on fibrillation inhibition (Supporting Information, Figure S11). However, the ability of AMP 1 to disrupt the fibril structure is unique, which is possibly linked to the presence of both positive and negative charges that facilitates balanced interactions with amyloid fibrils. Under agitation, the distinct surface chemistry of AMP 1 enables it to exert both attractive and repulsive forces on the fibrils, leading to greater destabilization and fragmentation. This fragmentation is further enhanced by the generation of ROS, which contribute to the oxidative degradation of the fibrils (Scheme 1). Our previous studies have shown that amyloid fibrils can induce ROS generation under mechanical stress via piezocatalytic activity, with this effect being amplified in the presence of plasmonic or magnetic nanoparticles.^52^ In the present study, the piezocatalytic generation of ROS during agitation likely plays a significant role in the accelerated degradation of the fibrils. The data also suggest that combined ultrasound and agitation lead to faster fibril disintegration compared to agitation alone, highlighting the synergistic effect of mechanical stress and ROS generation. These results indicate that the AMP 1 nanoparticles not only destabilize the fibrils through their surface interactions but are also capable of disintegrating the fibrils by promoting oxidative degradation. In contrast, CAT 1 nanoparticles, which carry only a positive charge, appear to be less effective at disrupting the fibrils. Their tendency to aggregate into larger nanoparticle clusters likely reduces their ability to interact efficiently with the fibrils, leading to insufficient fibril disintegration. Similarly, ANI 1 nanoparticles, which are negatively charged, might not interact as effectively with the fibrils, leading to less efficient disintegration.

## Conclusions

This study highlights the critical role of nanoparticle surface chemistry in modulating interactions with amyloid fibrils, which are key pathological features in neurodegenerative diseases such as Alzheimer’s disease (AD). Among the surface-engineered iron oxide nanoparticles tested, ampholytic nanoparticles—bearing both cationic and anionic surface groups—exhibited the strongest binding affinity and the most effective disintegration of mature amyloid fibrils. Mechanistic studies revealed that these ampholytic nanoparticles disrupt fibrils more efficiently under mechanical agitation through a combination of attractive and repulsive forces with the controlled generation of ROS. Furthermore, comparative studies with polymeric and quantum dot-based ampholytic nanoparticles confirmed that surface ampholyticity, not core composition, is the dominant factor in amyloid binding. The findings suggest that the nature of surface charge is critical for optimal performance and reduced ampholytic charge diminishes disintegration efficiency. Collectively, our results establish that ampholytic nanoparticles represent a broadly applicable and modular platform for targeting and dismantling amyloid fibrils, offering therapeutic promise for amyloid-associated neurodegenerative conditions.

## Supplementary Material



## Notes

Zeta potentials, coating strategy, colloidal stability study, TEM images of nanoparticle–fibril interaction study, MTT assay, NBT assay, and quantum dot (QD)–fibril interaction (PDF)
