# Supplementary material for: Nanoparticles with Ampholytic Surfaces for Binding and Disintegration of Amyloid Fibrils
Source: ACS Cent Sci. 2025 Jul 2;11(7):1218–29. doi: 10.1021/acscentsci.5c00519 (PMC12291134; doi:10.1021/acscentsci.5c00519)
Supplement: Supplementary file 1 [file oc5c00519_si_001.pdf]

Supporting Information

for

Nanoparticles with Ampholytic Surfaces for Binding and Disintegration of Amyloid Fibrils

Suman Mandal,[1,2] Minh Dang Nguyen,[1] Nikhil Ranjan Jana,[2,*] and T. Randall Lee[1,*]

[1]*Department of Chemistry and the Texas Center for Superconductivity, University of Houston, Houston, Texas 77204-5003, United States*

[2] *School of Materials Science, Indian Association for the Cultivation of Science, Kolkata 700 032, India*

Correspondence: trlee@uh.edu; camnrj@iacs.res.in

Table S1. Zeta potentials of the nanoparticles in different buffer conditions.

Sample	Zeta potential (mV)		
	**pH 4.5**	**pH 7.4**	**pH 9**
CAT 1	+ 16 ± 2	+ 10 ± 2	+ 8 ± 1
AMP 1	+ 3 ± 1	- 2 ± 1	- 5 ± 2
ANI 1	- 6 ± 3	- 7 ± 2	- 10 ± 1
AMP 2	+ 2 ± 1	- 1 ± 3	- 2 ± 2
ANI 2	- 8 ± 3	- 9 ± 2	- 11 ± 2
AMP 3	+ 2 ± 2	+ 1 ± 1	+ 3 ± 1
AMP 4	+ 4 ± 1	+ 1 ± 3	+ 2 ± 3

Table S2. Zeta potentials of the synthesized amyloid fibrils.

Protein/fibril	Purification procedure	Zeta potential (mV)	
		**H_2O**	**pH 7.4 buffer**
Lysozyme protein		+ 2 ± 1	- 3 ± 1
Lysozyme fibril	Centrifugation & redispersion	+ 8 ± 2	+ 3 ± 1
Lysozyme fibril	dialysis	- 3 ± 2	+ 1 ± 2

Scheme S1. Reverse microemulsion based phase transfer and polyacrylate coating approach in making water soluble Fe_3O_4 nanoparticles with modular surface chemistry. Nanoparticles and acryl monomers are dispersed within a reverse micelle composed of Igepal and cyclohexane. Initiation of a polyacrylate coating takes place under an inert atmosphere with the addition of persulfate. After a reaction period of 1-2 hours, the resulting polyacrylate-coated nanoparticles are precipitated using ethanol, subsequently rinsed, and finally dissolved in water.

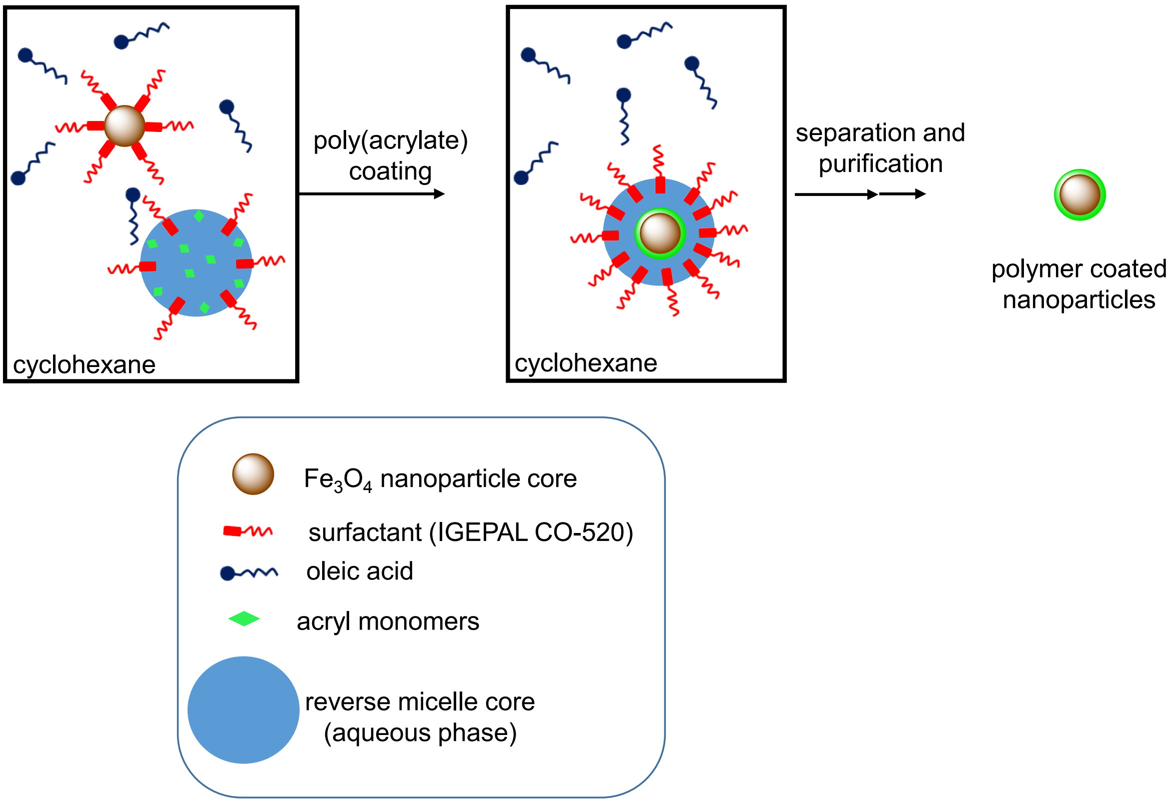

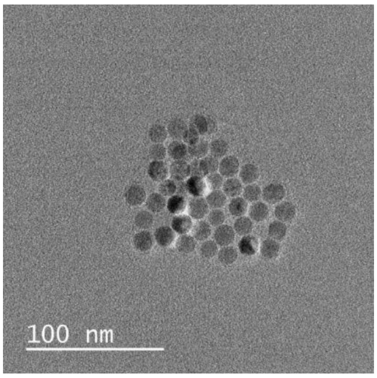

Figure S1. TEM image of ~16 nm anionic 2 (ANI 2) Fe_3O_4 nanoparticles.

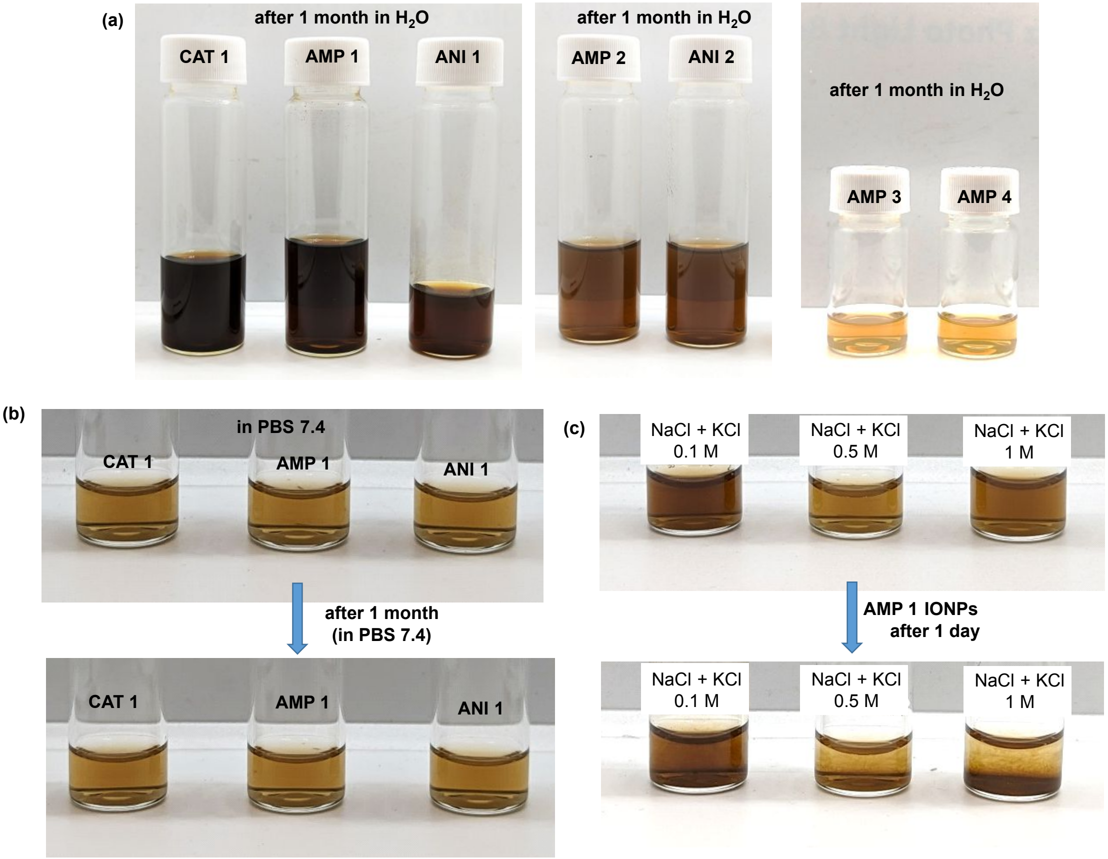

Figure S2. Images of water soluble IONPs and their colloidal stability in different salt and buffer conditions: (a) Displays the water-soluble nanoparticles utilized in the study (images captured after one month). (b) Shows colloidal stability in buffer PBS pH 7.4. (c) Shows colloidal stability in different salt conditions.

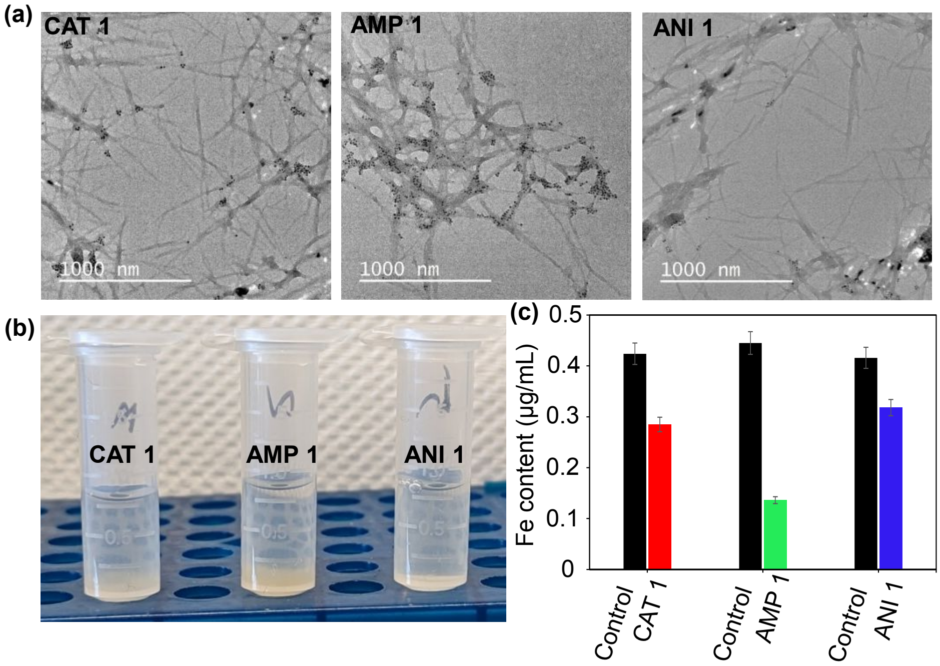

Figure S3. (a) IONP-fibril interaction analysed using a modified drop-casting method. (b) Digital images of colloidal solutions after 30 minutes of IONP-fibril incubation, followed by centrifugation at 1000 rpm to precipitate the fibrils along with the associated IONPs. (c) ICP-MS analysis of the supernatant from the above experiment to measure iron content.

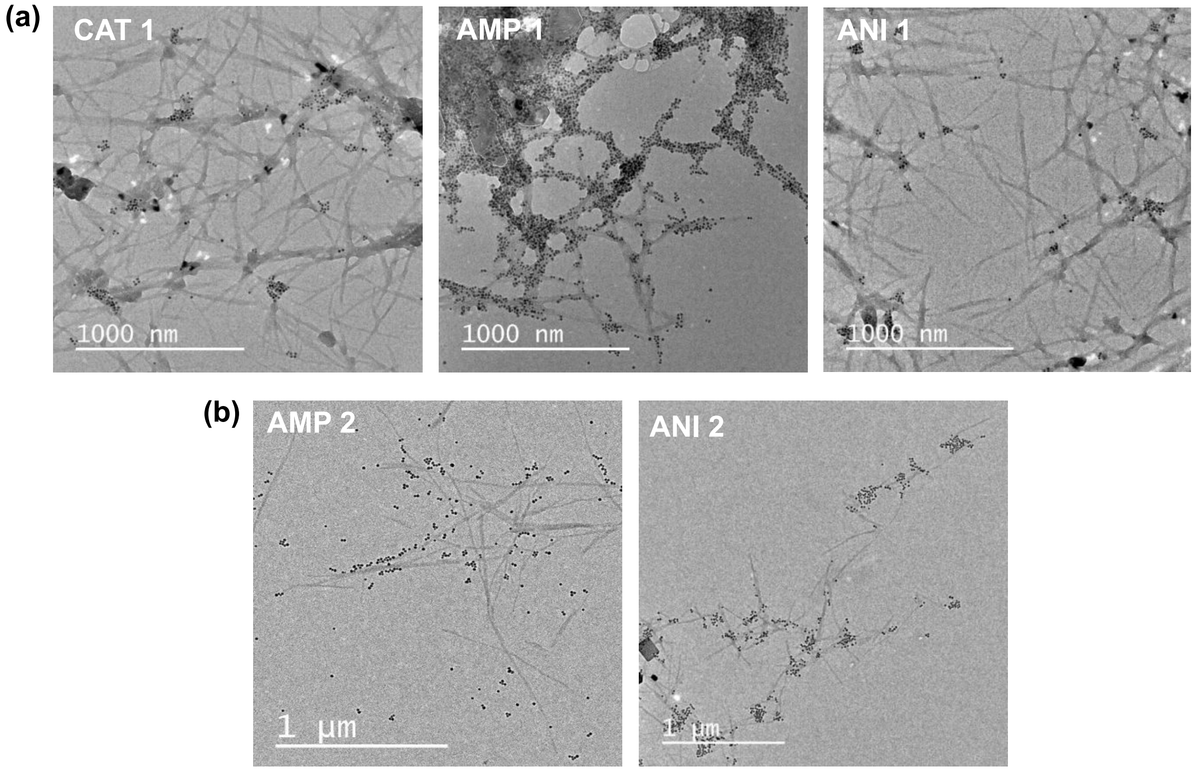

Figure S4. Interaction study between IONPs and the lysozyme amyloid fibrils purified by precipitation-redispersion method. (a) Showing the CAT 1, AMP 1 and ANI 1 along with LF. (b) Showing AMP 2 and ANI 2 with the fibrils.

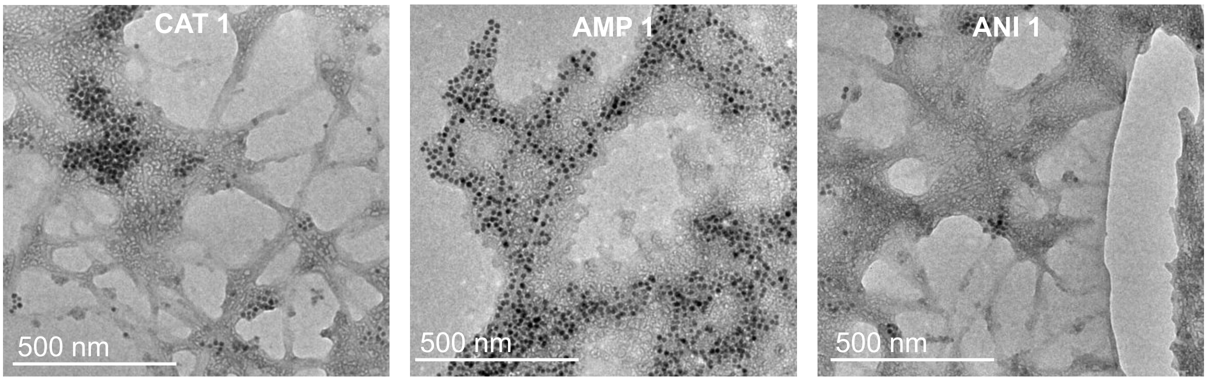

Figure S5. TEM images showing the interaction of surface-modified nanoparticles (AMP-1, CAT 1, ANI 1) with lysozyme fibrils in DMEM culture medium. AMP-1 nanoparticles exhibit stronger fibril association.

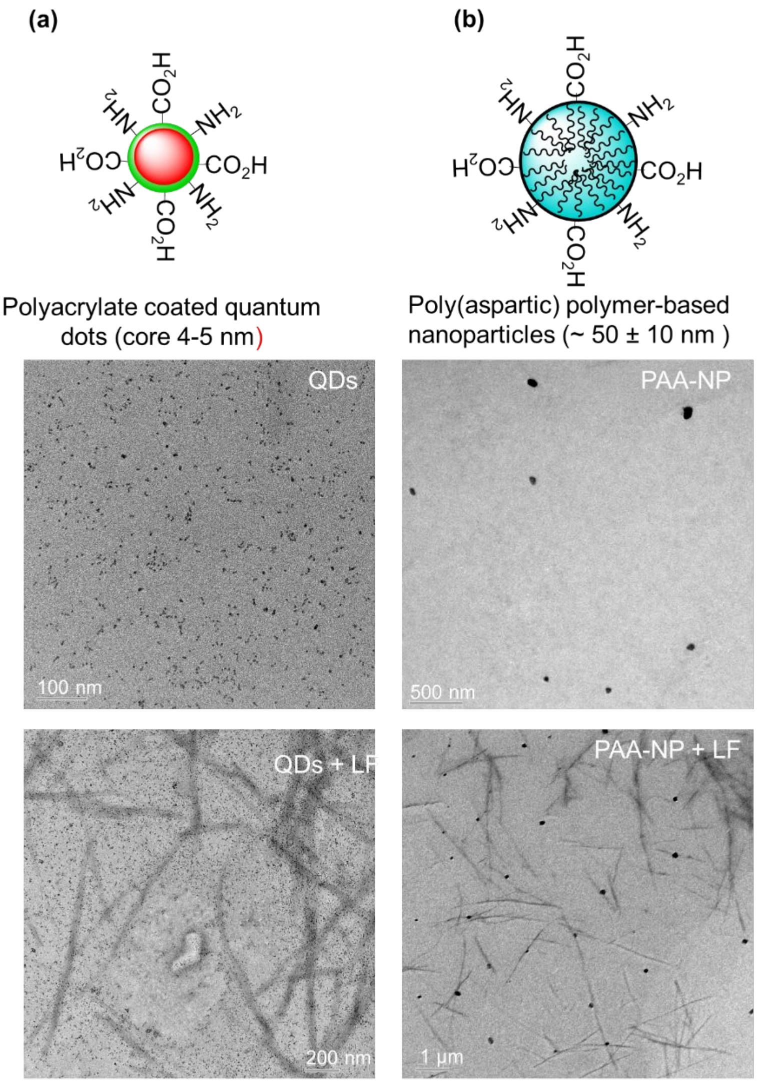

Figure S6. TEM images showing the binding of amyloid lysozyme fibrils (LF) with ampholytic nanoparticles of varied core materials: (a) polyacrylate-coated ampholytic quantum dots and (b) functionalized poly(aspartic acid)-based polymeric nanoparticles exposing primary amines and carboxylates on the surface (size ~ 50 ± 10 nm). Despite differing cores, both nanoparticle types exhibit fibril association, highlighting the dominant role of ampholytic surface chemistry mediating amyloid interactions.

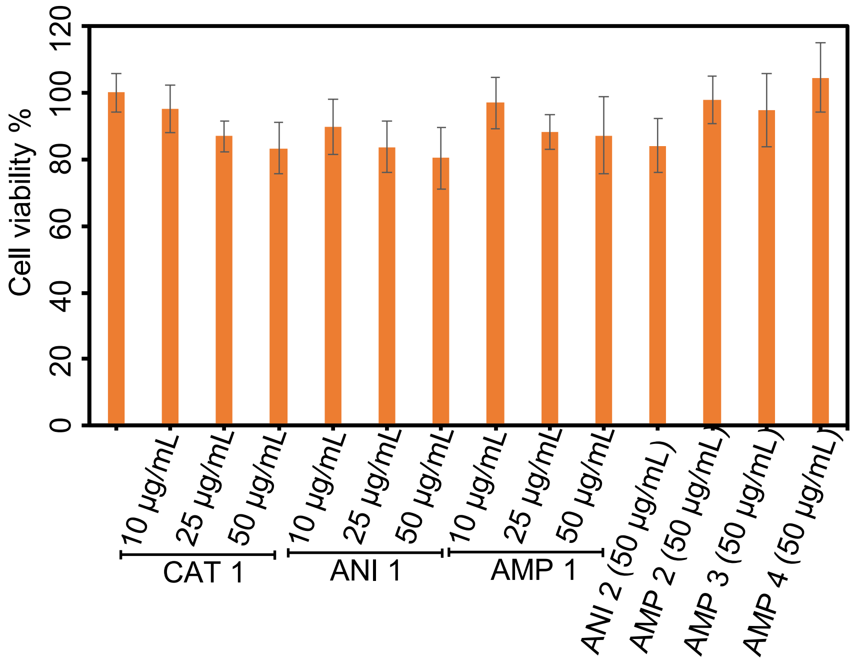

Figure S7. MTT based cytotoxicity assay in HT22 cell line.

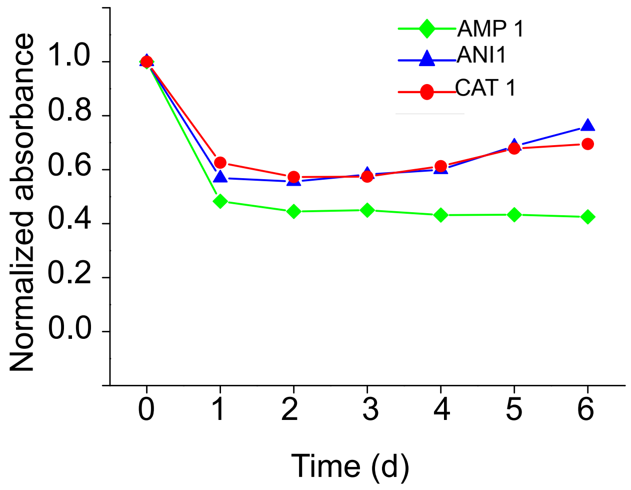

Figure S8. NBT assay has been done throughout the fibril disintegration study. The absorbance taken once in a day during the experiment.

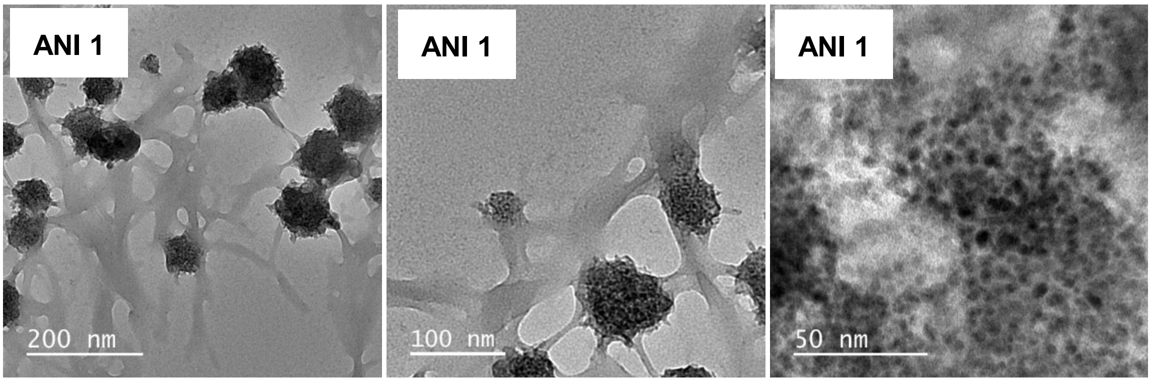

Figure S9. Fibril disintegration in presence of ANI 1 nanoparticles: TEM images taken in different magnification to see the aggregated chunks.

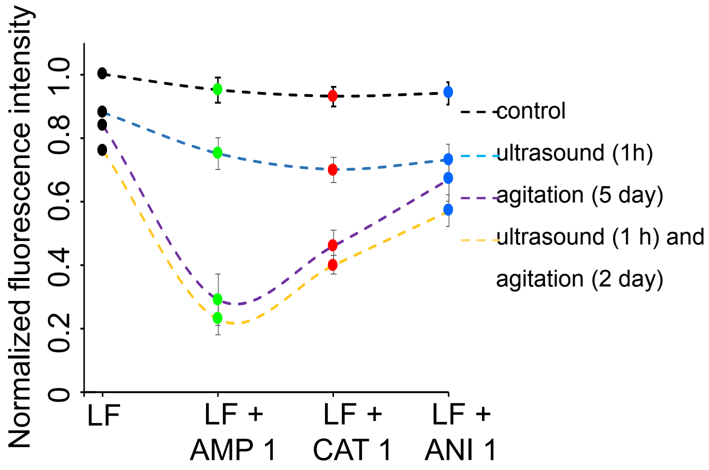

Figure S10. Fibril disintegration study (end point ThT assay) in presence of the IONPs under various conditions. The control set represents fibrils incubated with IONPs without the application of external force.

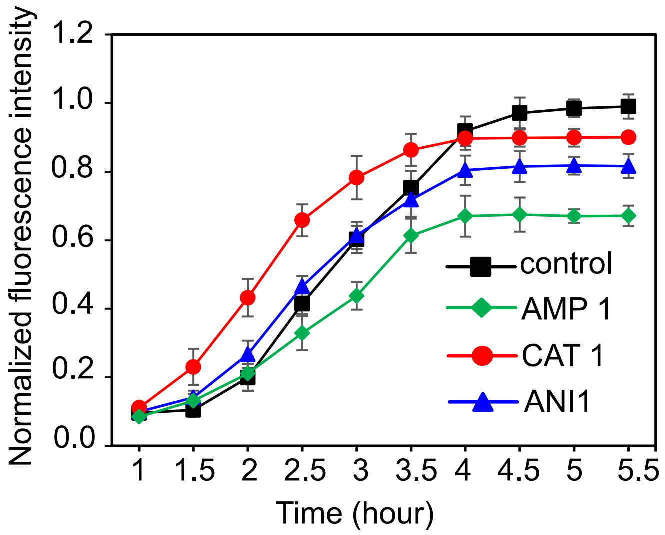

Figure S11. Fibrillation inhibition study (ThT assay) in presence of the IONPs. The control set represents lysozyme protein incubated without any IONPs.
